# Application of a New, Energy-Based ΔS* Crack Driving Force for Fatigue Crack Growth Rate Description

**DOI:** 10.3390/ma12030518

**Published:** 2019-02-09

**Authors:** Grzegorz Lesiuk

**Affiliations:** Faculty of Mechanical Engineering, Department of Mechanics, Materials Science and Engineering, Wroclaw University of Science and Technology, PL-50370 Wrocław, Poland; Grzegorz.Lesiuk@pwr.edu.pl; Tel.: +48-71-320-3919

**Keywords:** fatigue crack growth, mean stress effect, *J*-integral, energy approach, generalized Paris’ Law, crack growth rate, *R*-ratio

## Abstract

This paper presents the problem of the description of fatigue cracking development in metallic constructional materials. Fatigue crack growth models (mostly empirical) are usually constructed using a stress intensity factor Δ*K* in linear-elastic fracture mechanics. Contrary to the kinetic fatigue fracture diagrams (KFFDs) based on stress intensity factor *K*, new energy KFFDs show no sensitivity to mean stress effect expressed by the stress ratio *R*. However, in the literature there is a lack of analytical description and interpretation of this parameter in order to promote this approach in engineering practice. Therefore, based on a dimensional analysis approach, Δ*H* is replaced by elastic-plastic fracture mechanics parameter—the Δ*J-*integral range. In this case, the invariance from stress is not clear. Hence, the main goal of this paper is the application of the new averaged (geometrically) strain energy density parameter ΔS* based on the relationship of the maximal value of *J* integral and its range Δ*J*. The usefulness and invariance of this parameter have been confirmed for three different metallic materials, 10HNAP, 18G2A, and 19th century puddle iron from the Eiffel bridge.

## 1. Introduction

Fatigue and fatigue cracking are the two main (more than 70% of all failures) phenomena responsible for the process of destroying steel structures. The fatigue crack growth phase is an essential process for the long-term operating structures subjected to cyclic loading. A fatigue crack may be formed either as a result of the accumulation of fatigue damage (intrusions and extrusions) or as a result of manufacturing processes. The appearance of fatigue cracks can also be the result of unfavorable operating conditions. The mere fact that a crack exists does not necessarily (anymore) mean that such an element (still referred to as metallic construction materials) has to be eliminated from service. The period of precritical fatigue crack growth can be expressed in a general way using an integral:

(1) $$N_{cr}=\int_{a_{o}}^{a_{cr}}\frac{da}{f(\sigma_{ext},a,P_{fc},Y,R)}$$

where: *N_cr_* is precritical fatigue crack growth period, *a_o_* is the crack length, *a_cr_* is the critical crack length, *σ_ext_* is the external load, *P_fc_* is the fracture mechanics parameter, the so-called crack driving force (CDF), *Y* is the geometric constraint, and *R* (*σ_min_/σ_max_*) is the stress ratio. 

In the case of static loads, it is crucial to determine the critical load that triggers the avalanche development of the crack or to look for the critical length of the crack at which the element will continue to carry the assumed load. For safety reasons, an important issue is to determine the subcritical time of fatigue crack development under cyclic loading condition. According to the above, it is necessary to solve Equation (1) with known boundary conditions. However, in this case, it is crucial to provide a fatigue crack growth law that is as robust as possible. In the 1960s, Paris [1] correlated a quantity derived from classical fracture mechanics—Δ*K* stress intensity factor range (SIF)—with the fatigue crack growth rate from experimental data. Paris proposed this relationship in the form of a power-law function—known in the literature as the Paris law:

(2) $$\frac{da}{dN}=C{\left(\Delta K\right)}^{m}$$

**Figure 1:**
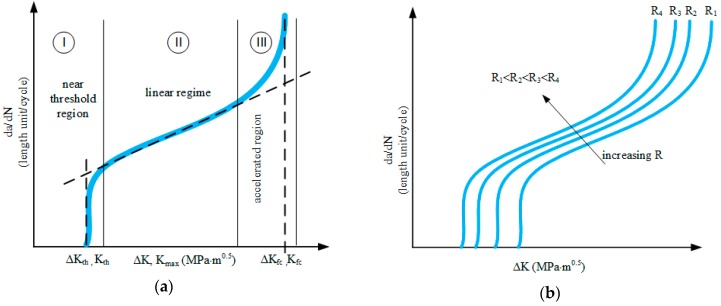
Schematic Kinetic Fatigue Fracture Diagram (KFFD): (**a**) sigmoidal shape of the fatigue crack growth rate (FCGR) curve; (**b**) the scheme of the stress *R*-ratio impact on the FCGR curves (*R*_4_ > *R*_3_ > *R*_2_ > *R*_1_).

Engineers are predicting the life of an element with a defect using various analytical and numerical techniques (including FEM and BEM). In Equation (2) *C, m* are constants determined from the Kinetic Fatigue Fracture Diagram (KFFD) presented in Figure 1. Δ*K* is related to the range of load changes Δ*K = K_max_ − K_min_*, corresponding successively to the range of external load changes. 

The constant *m* in the Paris’ law model is related to the angle of inclination of the experimental data straight line (Figure 1a), while the constant *C* is the ordinate at the intersection of the extension straight line from Area II. Generally speaking, the typical graph of fatigue cracking kinetics is divided into three areas (Figure 1a). Area I is the domain of the so-called low crack growth rate, i.e., from 0 to approximately 10^−9^ m/cycle, while Area II corresponds to the straight line of average fatigue crack growth rate in the range 10^−9^–10^−6^ m/cycle—so called Paris regime. Area III is the domain of high fatigue crack growth rate, i.e., above 10^−5^ m/cycle. These ranges are contractually accepted as they depend on the material, its properties, the environment, and the load itself. The range (I) is limited on the left by the asymptote corresponding to the threshold Δ*K_th_* value—the threshold range of stress intensity coefficient below which fracture does not propagate or propagates at an insignificantly low speed. The development of cracking ends when the stress factor reaches the critical value Δ*K_fc_*, above which cracking propagates unstable. The effect of the stress ratio *R* factor is schematically represented as in Figure 1b. For higher *R*-factors, the Δ*K_th_* threshold value, which triggers the fatigue cracking process, is lower, and all da/dN-Δ*K* curves are shifted. Therefore, the adaptation of Equation (2), taking into account the effect of the cycle asymmetry coefficient, is a major topic in Fatigue Fracture Mechanics (FFM). One of the well known is the solution proposed by Forman [2]:

(3) $$\frac{da}{dN}=\frac{C{\left(\Delta K\right)}^{m}}{(1-R)K_{C}-\Delta K}$$

The *K_c_* quantity in Equation (3) is the fatigue fracture toughness (or critical stress intensity factor) for the given load conditions. In the event of difficulties in its establishment, it is very often replaced by known *K_IC_*. Accurate determination of constants *m* and *C* requires knowledge of crack speed courses for different cycle asymmetry coefficients. Another proposal is well-known as Walker's law [3]:

(4) $$\frac{da}{dN}=\frac{C_{w}}{{\left(1-R\right)}^{n_{w}}}{\left(\Delta K\right)}^{m_{r}}$$

The constant *C_w_* is determined here experimentally for different values of *R*. For *R* = 0 this constant is equivalent to the Paris’ constant *C*. The exponent *m_r_* is treated as a constant, similarly *n_w_*—is obtained from experimental data. It is also determined by the extrapolation of data from kinetic fatigue fracture diagrams constructed for different *R*-ratios. 

The influence of the cycle asymmetry coefficient strongly determines the analytical description of the fatigue gap closing process noticed by Wolf Elber [4]. The model covering the above issues is the model known in the literature on the subject of Forman-Mettu [5]:

(5) $$\frac{da}{dN}=C{\left[\left(\frac{1-f}{1-R}\right)\Delta K\right]}^{n}\frac{{\left(1-\frac{\Delta K_{th}}{\Delta K}\right)}^{p}}{{\left(1-\frac{K_{\max}}{K_{c}}\right)}^{q}}$$

In the Forman-Mettu model expressed in Equation (5), *c, n, p*, and *q* are experimentally determined constants, and *f* is associated with the function of crack opening. The form of this function can be determined based on, e.g., FITNET procedures [6]. However, the application of this model is not easy—mainly due to a large number of experimentally determined constants. 

**Figure 2:**
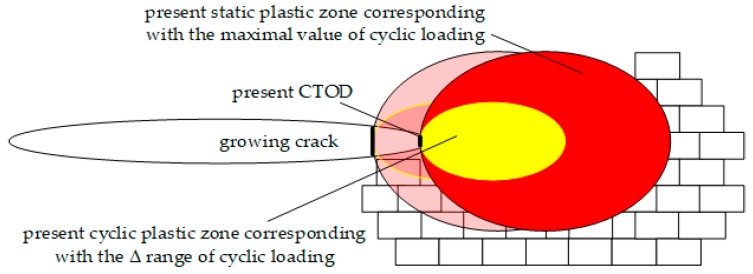
Schematic representation of plastic zones ahead of a fatigue crack tip with the marked Crack Tip Opening Displacement CTOD—*δ*.

A different group of attractive fatigue fracture models are the mathematical models based on the relationship of low cycle fatigue (LCF) parameters with the fatigue crack propagation rate. Fatigue crack growth is considered an elementary act in the local plastic zone of fracture including two different areas: static, corresponding to the maximum value of load in the cycle, and cyclic, corresponding to the amplitude of load. This concept is presented in Figure 2. Several authors [7,8,9,10,11,12,13,14] proposed excellent relationships between LFC (Low Cycle Fatigue) and FCGR (Fatigue Crack Growth Rate) data. However, in all cases, the presented models work well for the maintained *R*-ratio. It is more likely that the main reason of the mathematical collapse in *R*-ratio invariance of the fatigue crack growth description is associated with the fact that the crack driving force still depends only on the Δ*K* range or on the maximum value of *K*. 

Kujawski [15,16] proposed a new, crack driving force for a Δ*K* description of FCGR—the geometrical mean value of positive Δ*K* and *K_max_*. The next development of the proposed model was the introduction of the weighting exponent *α* [17,18]:

(6) $$K^{*}={\left(\Delta K^{+}\right)}^{1-\alpha }\cdot K_{\max}^{\alpha }$$

According to the above, it is worth underlining an excellent contribution of Kujawski’s model into the force approach combaning positive part of the stress intensity factor range Δ*K-*Δ*K^+^* and *K*_*max*_. However, the meaning of the α parameter (ranged from 0 to 1) is debatable and in each case should be calibrated from *da/dN-*Δ*K* curves. 

Therefore, the main aim of this work is to propose a new, crack driving force parameter with a good physical interpretation responsible for the fatigue fracture process without *R*-ratio influence. 

Alternative methods of describing the kinetics of fatigue cracking are also being searched in order to eliminate the problem of avoiding the *R*-ratio effect. Research conducted by the author shows that it is possible to obtain such a dimensional base for KFFD, in which the description of fatigue cracking kinetics will not depend on the stress ratio. Energy as a dimensional quantity combining the dimensions of “force” and “displacement” seems to be naturally predestined to describe the kinetics of cracking. Many of the hypotheses concerning both fatigue and the description of fatigue cracking are based on energy irreversibly dissipated in each cycle of the load spectrum [19,20]. The dissipated energy accumulated in the material can be recorded as a hysteresis loop during the test. Determination of the subcritical period of fatigue crack growth requires the application of the first principle of thermodynamics. Assuming an infinite solid body model subjected to sinusoidal alternating external loads with a central part-thru, this balance can be formulated as follows [21,22,23,24]:

(7) $$A+Q=W+K_{e}+\Gamma$$

In Equation (7), *A* represents the work of external loads after *N* cycles, *Q* represents the heat supplied to the body during loading, and *W* is the deformation energy after *N* cycles. The kinetic energy of the body is marked as *K_e_*. *Γ* is the damage energy necessary for fatigue crack growth increment. When formulating the energy balance expressed in Equation (7), according to [23], the quantities *A, Q, W*, and *K_e_*, are referred to unit of thickness. It is also assumed that the fatigue crack will grow slowly during each cycle so that the kinetic energy and heat exchanged during this process can be disregarded (i.e., for low loading frequencies). After the differentiation of Equation (7) and simplifications, the energy balance can be represented by

(8) $$\frac{\partial A}{\partial N}=\frac{\partial W}{\partial N}+\frac{\partial \Gamma }{\partial N}$$

However, damage energy *Γ* can be expressed as a sum of static and cyclic components:

(9) $$\Gamma =W_{c}+W_{s}$$

The energy of the static (monotonic) component of cyclic plastic deformations *W_s_*, corresponding to the maximal value of external loading, is considered as the maximum value of the dissipation of the static energy activating the fracture process without the energy of cyclic deformation changes (*W_c_* = 0) [24]. *W_c_* corresponds to the dissipated energy during cyclic loading. For fatigue crack growth, both quantities are equally important. Hence, the final form of the fatigue crack growth surface can be expressed as

(10) $$\frac{\partial S}{\partial N}=\frac{\partial W_{c}/\partial N}{\partial (\Gamma -W_{s})/\partial S}$$

(11) $$dS/dN=\frac{\alpha W_{c}^{\left(1\right)}}{\sigma_{plf}\varepsilon_{fc}(1-K_{I\;\max}^{2}/K_{fc}^{2})}$$

Thus, the proposed crack driving force Δ*H* (expressed in J/m^2^) is equal:

(12) $$\Delta H=\frac{W_{c}^{\left(1\right)}}{B(1-K_{I\;\max}^{2}/K_{fc}^{2})}$$

The Paris-like model can be represented by

(13) $$da/dN=A{\left(\Delta H\right)}^{k}$$

In the above equations, *α* is a constant dimensionless factor, *S* is the fatigue crack area, *σ_plf_* is the cyclic yield point*,* *ε_fc_* is the critical strain value under cyclic conditions, and Δ*H* is the new energy parameter—the crack-driving force. In Equation (13), *k* should be equal to 1 (based on dimensional analysis approach). However, it is also reported in [22] that *k* varies from 0.87 to 1.38 for different ductility levels of the tested materials. On the other hand, in the original approach [24], this parameter strongly depends on the hysteresis loop area measured in the load line. It is more likely that this approach seems to be correct from a physical point of view but strongly depends on the experimental techniques of the registered dissipated energy. The problem of measuring dissipated energy regarding the global-local energy approach is widely discussed in [19]. There are no doubts that the energy approach supported by numerical methods is a powerful tool in a proper description of FCGR and residual lifetime estimation based on a rather physical not an empirical model. So far, no attempts have been made to analyze the Δ*H* characterization of the parameter, to link it with well-known parameters from classical fracture mechanics, i.e., CTOD or *J*-integral. This fact explains, among other reasons, the relatively low acceptance of energetic models in engineering practice. From the physical point of view, the other well interpretable quantity with this physical dimension Δ*H* is the integral *J*. Moreover, contemporary numerical and full field experimental methods allow determining the integral *J* for advanced materials and loading cases in an efficient way. Many times in the range of linear fracture mechanics, integral *J* allows one to determine stress intensity factors where there are no closed-form analytical solutions. This has been demonstrated in the author’s and co-author’s works [25,26,27,28] devoted to mixed mode fatigue crack growth description. However, it is worth noting that the number of kinetic energy models based on integral *J* is still negligible. Dowling and Begley [29] were the pioneers in describing the kinetics of fatigue cracking [29] using the cyclic *J* integral concept. At a later stage, Dowling [30] demonstrated the independence of integral *J* as a CDF (Crack Driving Force) from the geometry of samples, which encouraged the stability of this size in the description of the kinetics of fatigue curing. The path independence in applications to fatigue crack growth problems is also proven in many papers [31,32,33,34]. However, also in this case, the full invariability with respect to the *R*-ratio coefficient is not always obtained by substitution of the crack driving force from Δ*K* to the integral Δ*J*. As evidence, the experimental data for 18G2A and 10HNAP steels (according to standard Polish nomenclature) recorded in papers [35,36,37,38,39] using bended specimens in FCGR experiment in a linear and nonlinear fracture mechanics validity range.

Independently from Rozumek’s results [36,37,38,39], Joyce et al. [40] reported for cast stainless steel equivalent to ASME SA-351CF8M a similar impact of *R*-ratio in elastic-plastic cyclic Δ*J*-crack driving obtained from standardised compact tension (CT) specimens. On the other hand, there are no doubts that the *J*-integral crack driving force can be applied where the validity of the linear elastic fracture mechanics is limited, so the Δ*K* approach is debatable. However, experimental observations [29,30,31,32,33,34,35,36,37,38,39,40] have confirmed the fact that the *J*-integral approach does not entirely solve the problem of the mean stress effect in the description of the FCGR. On the other hand, many analytical models seem to confirm the fact that the solution in the unification of the fatigue cracking process should be sought not so much in the amplitude of the force driving the crack but in the mutual combination of maximum CDF values and CDF amplitude.

**Figure 3:**
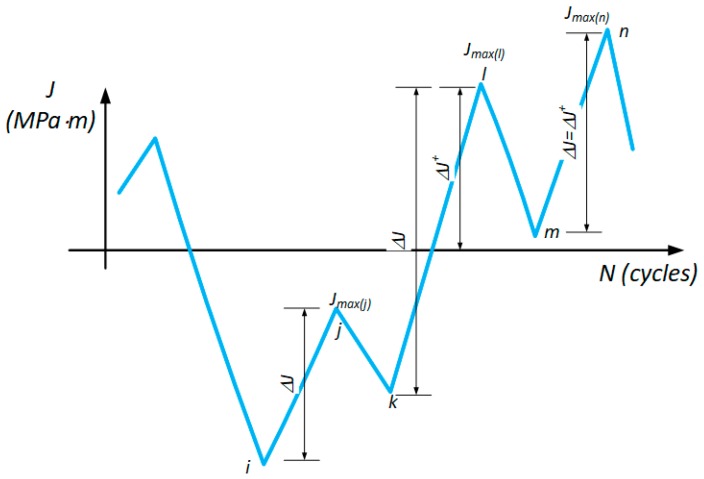
Schematic representation of the ΔS* components during cyclic loading.

Considering the above, the previously formulated basics of energy modeling allowed for the determination of the relation between fatigue fracture rate and energy parameters. Details of the dimensional analysis modeling [41,42] are available in [21,22,23,24,43,44]. In these works, it was demonstrated that the required energetic parameter with the physical dimension (J/m^2^) is responsible for the fatigue fracture process. As it is based on the energy measured in the form of a local hysteresis loop, the ambiguities with multi-axis loads and geometries of the specimens did not allow for its proper application. On the other hand, the concept initiated by Kujawski [16] for the elastic parameter Δ*K* seems to be worth considering when applying for elastic-plastic states. From experimental works and methods of energy accumulation [45], it can be indicated that schematically variable average load connects Δ*J* and *J_max_*. During fatigue crack growth, as is shown in Figure 3, under a different load ratio *R*, in the case of load paths from point *i* to *j* (negative maximal load value), the damage accumulation cannot cause crack growth (no crack opening means that Δ*J^+^* is zero). For the load case from *k* to *l* (negative stress ratios), the crack is partially open (excluding the Elber crack closure phenomenon) in the positive part of the loading (Δ*J^+^* < Δ*J*). For the positive *R*-ratios (load path from *m* to *n*), the Δ*J^+^* is equal to Δ*J*. It is worth noting that, during a cyclic load, *J_max_* and Δ*J* are the values that bind the local stress intensity in front of the crack front—*J* plays the same role as *K* in the elastic-plastic fracture mechanics. Therefore, the geometric mean of Δ*J^+^* and *J_max_* is proposed as a new crack driving force candidate:

(14) $$\Delta S*=\sqrt{\Delta J^{+}\cdot J_{\max}}$$

## 2. Kinetic Fatigue Fracture Diagrams for 10HNAP, 18G2A, and the Eiffel bridge 19th-Century Puddle Iron

**Figure 4:**
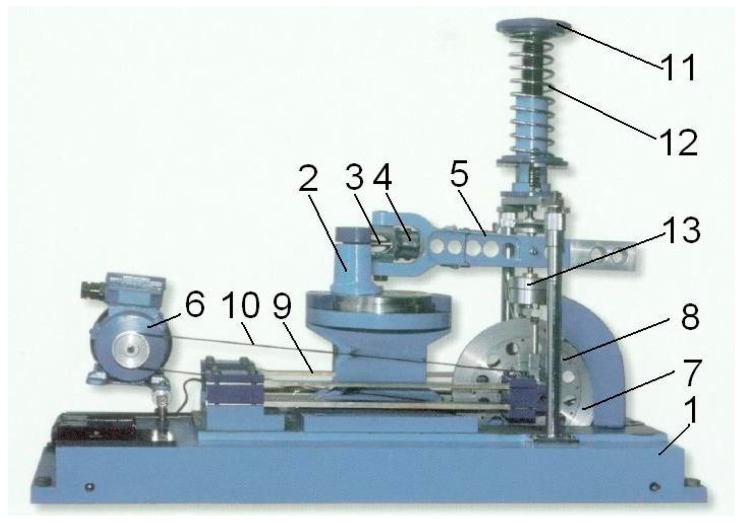
Fatigue testing machine MZGS-100: 1—bed, 2—rotational head with a holder, 3—specimen, 4—holder, 5—lever (effective length = 0.2 m), 6—motor, 7—rotating disk, 8—unbalanced mass, 9—flat springs, 10—driving belt, 11—spring actuator, 12—spring, 13—hydraulic connector [36].

For experimental verification, the published fatigue crack growth data (for steel 10HNAP and 18G2A) based on the Δ*J* integral range [35,36,37,38] was used. In Rozumek’s papers [35,36,37,38], the test fatigue crack growth experiment based on elastic-plastic fracture mechanics was performed on the fatigue test stand MZGS-100 (Figure 4), enabling cyclically variable and static (mean) loading. 

**Figure 5:**
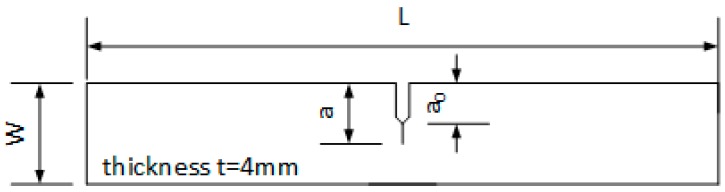
Scheme of the specimen subjected for the bending test in Gasiak & Rozumek [35] experiment, *L* = 120 mm, *W* = 20 mm, notch length *a*_0_ = 5 mm (notch tip angle 60°, root radius *ρ* = 5 mm), *a*—fatigue crack length.

In an experimental campaign (based on Δ*J* parameter as a crack driving force), a bent beam (Figure 5) made from 10HNAP and 18G2A steel was used. The specimens subjected to bending had an external unilateral sharp notch of 5 mm in depth, with the rounding radius *ρ* = 0.5 mm. The specimen dimensions were length *L* = 120 mm, width *W* = 20 mm, and thickness *t* = 4 mm. The scheme of the specimen is presented in Figure 5. 

In the cited experimental campaign, the total Δ*J* parameter [24,35,36] was considered as a sum of the elastic and plastic components of the cyclic Δ*J* parameter [24]:

(15) $$\Delta J=\Delta J_{e}+\Delta J_{p}=2\pi Y^{2}a\left(\int_{0}^{\Delta \varepsilon_{e}}\sigma d\varepsilon_{e}+\int_{0}^{\Delta \varepsilon_{p}}\sigma d\varepsilon_{p}\right)$$

In Equation (15) Δ*J_e_* represents elastic part of Δ*J* integral range, Δ*J_p_* – plastic part, *Y*—geometric constraint, *ε_e_*—elastic strains, *ε_p_*—plastic strains measured in the vicinity of a crack tip. The tests were performed on the fatigue test stand MZGS-100 (Figure 3) with the loading frequency 29 Hz and the maximal bending moment was equal: *M_a_* = 15.64 nm under the Mode I condition. Three different *R*-ratios were considered; *R* = 0, *R* = −0.5, *R* = −1. During the experiments, the crack length was observed using optical methods. All experimental details can be found in Rozumek’s papers [36,37,38,39]. After experiments, the elastic-plastic *J* integral was calculated using boundary element methods (BEM) and FRANC3D software for fracture mechanics analysis. The detailed experimental-numerical procedure is described in [36,37,38,39]. Finally, the elastic-plastic, kinetic fatigue fracture diagrams were constructed based on the *J*-parameter.

**Figure 6:**
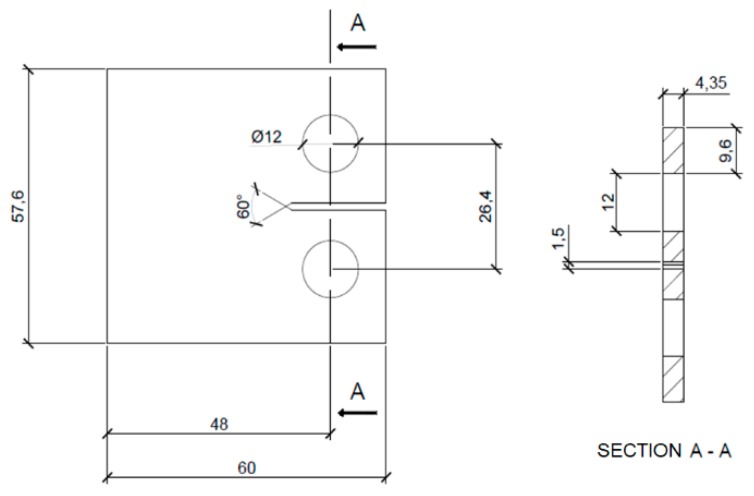
Compact Tension (CT) specimen scheme and dimensions for the Mode I FCGR experiment (puddle iron, all dimensions in mm).

As an alternative, for the ΔS* crack driving force, the kinetic fatigue fracture diagrams were parallel-constructed. The FCGR experiment for the Eiffel bridge [46] puddle iron was designed and performed in accordance with the American standard ASTM E647 [47]. Scheme of the specimen is presented in Figure 6. The stress intensity factor (SIF) for the standardized Compact Tension (CT) specimen is described by [47]

(16) $$\Delta K=\frac{\Delta F}{B\sqrt{W}}f(a/W)$$

(17) $$f\left(\frac{a}{W}\right)=\frac{\left(2+\alpha )(0.886+4.64\alpha -13.32\alpha^{2}+14.72\alpha^{3}-5.6\alpha^{4}\right)}{\sqrt{{\left(1-\alpha \right)}^{3}}}$$

where *α* is the normalized crack length referred to the specimen width (*α = a/W*), *B* is the thickness of the specimen, *W* represents the specimen width, and ∆*F* is the applied force range. The crack length was monitored using compliance methods from the clip-gage mounted on additional knives on the front side of the specimen—CMOD (crack mouth opening displacement). 

For the energy fatigue crack growth description (puddle iron from the Eiffel bridge), only the elastic part of the Δ*J*-integral range was analyzed using a well-known relationship from linear-elastic fracture mechanics (with assumed plane stress conditions):

(18) $$\Delta J_{e}=\frac{\Delta K^{2}}{E}$$

**Figure 7:**
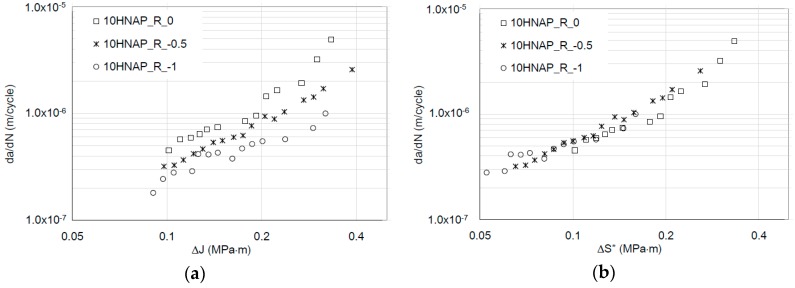
Fatigue crack growth curves for 10HNAP steel (**a**) based on the Δ*J* crack driving force (based on data from [37]) and (**b**) based on the new, averaged ΔS* crack driving force.

**Figure 8:**
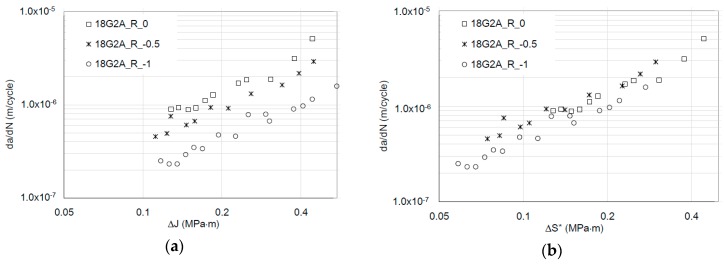
Fatigue crack growth curves for 18G2A steel (**a**) based on the Δ*J* crack driving force (based on data from [37] and (**b**) based on the new, averaged ΔS* crack driving force.

**Figure 9:**
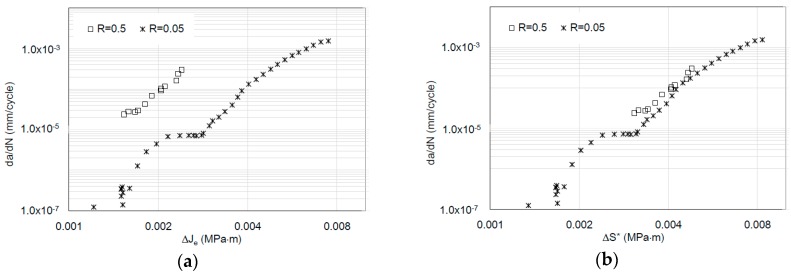
Fatigue crack growth curves for the puddle iron from the Eiffel bridge (**a**) based on the Δ*J* crack driving force and (**b**) based on the new, averaged ΔS* crack driving force.

The chemical composition of analyzed materials is presented in Table 1. Table 2 and Table 3 include both static and cyclic mechanical properties of the considered materials. Kinetic fatigue fracture diagrams for all materials are shown in Figure 7, Figure 8 and Figure 9.

As observed, the impact of R is noticeable in the case of the Δ*J* description in the FCGR diagrams. In the same way, a similar tendency in the case of the da/dN-*J_max_* description using the relationship between *J_max_* and Δ*J* can be demonstrated. However, in the case of an energy approach, the main concept assumed the representation of the fatigue crack growth rate using invariant kinetic fatigue fracture diagrams based on a new, crack driving force—ΔS*. ΔS* involves both Δ*J* and *J_max_* parameters. However, Δ*J* should be limited only for Δ*J*^+^. Of course, possible corrections can be done using an effective Δ*J^+^* range based on crack closure measurements. Recently, in [48,49], an efficient experimental method was described for in-situ *J*-integral calculation using DIC—digital image correlation. In the presented case, in order to analyze the invariance ability from stress ratio *R*, the crack driving force was examined by the simple statistical *R*^2^ data fitting using a Paris-like relationship, for Δ*J* and ΔS* crack driving forces:

(19) $$\frac{da}{dN}=A_{1}{\left(\Delta J\right)}^{n}$$

(20) $$\frac{da}{dN}=D{\left(\Delta S*\right)}^{p}$$

As was expected in each case, the new ΔS* crack driving force consolidated all experimental results for different *R*-ratios into one straight line in the Paris regime. Moreover, the Paris-like law data fitting is better in the case of the description of the FCGR curves using the newly proposed crack driving force. Statistical outputs of the R-square data fitting to the Equations (19) and (20) are presented in Table 4. According to the statistical analysis, in each case, the introduction of the ΔS* parameter caused a significant increase in data fitting for the Paris-like model expressed in Equation (20) in comparison to the model expressed in Equation (19). 

## 3. Conclusions

This article presents an understanding of dimensional analysis identifying the energy force driving the crack. In the course of this work, it was established that this value is the parameter of Δ*H* (J/m^2^) proposed by Szata [24]. In the absence of an unambiguous analytical interpretation of this parameter, it was replaced by the integral *J*, which did not allow avoiding the influence of average load in the form of *R*. A new CDF—ΔS*—based on the geometric mean of the maximum value of cyclic *J*-integral and its range Δ*J* has been proposed to describe the kinetics of fatigue cracking. Its suitability for three different engineering materials (10HNAP and 18G2A steels and puddle iron) has been tested. On the basis of the above considerations, the following conclusions may be formulated:

- In contrast to *J*, S* unambiguously describes the fatigue crack kinetics for 10HNAP and 18G2A steels in the range of non-positive stress ratio *R* (considering elastic-plastic conditions).
- In contrast to *J*, S* unambiguously describes the fatigue crack kinetics for 19th-century puddle iron from the Eiffel bridge in the range of positive stress ratio *R* (considering linear elastic fracture mechanics conditions).
- In each case, the description of KFFD including S* resulted in higher values of *R*^2^ data fitting coefficient for the power-law description of the FCGR in the Paris regime.
- A good physical interpretation of S* as opposed to *H* allows for its easy implementation into the numerical environment.

## Figures and Tables

**Table 1:** Chemical composition (in % by weight) of the tested materials.

Material	C	Mn	Si	P	S	Cr	Ni	Cu	Fe
10HNAP [37]	0.14	0.88	0.31	0.066	0.027	0.73	0.30	0.345	bal.
18G2A [36]	0.20	1.49	0.33	0.023	0.024	0.01	0.01	0.035	bal.
tested puddle iron	0.01	0.01	0.07	0.354	0.045	-	-	-	bal.

**Table 2:** Static mechanical properties of the analyzed metallic materials.

Material	Ultimate Tensile Strength UTS (MPa)	Yield Strength *σ_pl_/σ*_0.2_ (MPa)	Young Modulus *E* (GPa)	Poisson Ratio *ν* (-)	Fracture Toughness *J_IC_* (MPa·m)
10HNAP [37]	566	418	215	0.29	0.178
18G2A [36]	535	357	210	0.3	0.320
Eiffel Bridge puddle iron [46]	342	292	193	0.3	n/a

**Table 3:** Low cycle fatigue properties of the analyzed materials.

Material	Fatigue Strength Coefficient *σ_f_*’ (MPa)	Fatigue Ductility Coefficient *ε_f_*’ (-)	Fatigue Strength Exponent *b* (-)	Fatigue Ductility Exponent *c* (-)
10HNAP [37]	746	0.442	−0.080	−0.601
18G2A [36]	782	0.693	−0.118	−0.410
Puddle Iron Eiffel Bridge [46]	603	0.160	−0.078	−0.797

**Table 4:** Statistical analysis of data fitting for the Kinetic Fatigue Fracture Diagram (KFFD) based on Δ*J* and ΔS* (all *R*-ratios).

All Data from FCGR Tests	*R*^2^ da/dN-(Δ*J*) Equation (19)	*R*^2^ da/dN-(ΔS*) Equation (20)
10HNAP	0.73	0.94
18G2A	0.54	0.91
Puddle Iron from the Eiffel Bridge	0.64	0.97
